# A wild bumble bee shows intraspecific differences in sensitivity to multiple pesticides

**DOI:** 10.1098/rsos.250281

**Published:** 2025-06-25

**Authors:** Anna R. Tatarko, Rachel L. Vannette, Steven A. Frese, Anne S. Leonard

**Affiliations:** ^1^ Department of Biological Sciences, University of Nevada Reno, Reno, NV, USA; ^2^ School of Biological Sciences, University of Bristol, Bristol, UK; ^3^ Department of Entomology and Nematology, University of California Davis, Davis, CA, USA; ^4^ Department of Nutrition, University of Nevada Reno, Reno, NV, USA

**Keywords:** wild bees, gut microbiomes, pesticides

## Abstract

Wild pollinator declines are increasingly linked to pesticide exposure, yet it is unclear how intraspecific differences contribute to observed variation in sensitivity, and the role gut microbes play in the sensitivity of wild bees is largely unexplored. Here, we investigate site-level differences in survival and microbiome structure of a wild bumble bee exposed to multiple pesticides, both individually and in combination. We collected wild *Bombus vosnesenskii* foragers (*N* = 175) from an alpine meadow, a valley lake shoreline and a suburban park and maintained them on a diet containing a herbicide (glyphosate), a fungicide (tebuconazole), an insecticide (imidacloprid) or a combination of these chemicals. Alpine bees had the highest overall survival, followed by shoreline bees then suburban bees. This was in part explained by body size differences across sites and the presence of conopid parasitoids at two of the sites. Notably, site of origin impacted bee survival on the herbicide, fungicide and combination treatment. We did not find evidence of gut microbiome differences across pesticide treatment, nor a site-by-treatment interaction. Regardless, the survival differences we observed emphasize the importance of considering population of origin when studying pesticide toxicity of wild bees.

## 1. Introduction

Bumble bees and other wild bees are vital pollinators of crop plants and wildflowers in North America, often outperforming managed species like honey bees [1,2]. Due to their agricultural and ecological importance, bumble bee population declines have gained global attention [3–7] which have been linked to a combination of human-induced stressors. Key among these is the widespread use of insecticides, fungicides, and herbicides [8,9]. Agricultural chemicals can be found in the tissue of a broad array of plant taxa and have become ubiquitous in agricultural settings as well as urban, suburban, and even conservation areas [10–12]. These chemicals’ detrimental impact on bee survival, reproduction and even behaviours can scale to have population-level impacts which persist for generations post-exposure [13–19]. Moreover, pesticides are rarely observed in isolation and are often detected as combinations of different chemical classes (i.e. insecticides, fungicides, and herbicides) in a complex chemical cocktail [10,11,20,21]. Understanding synergistic effects of pesticides on bee health is therefore an active area of study [19,22–26].

Beyond direct physiological harm, these chemicals may indirectly affect bee health by disrupting the gut microbiome. The bumble bee gut harbours a simple community of microbial symbionts that aid in digestion, growth, protection against parasites and pathogens, and detoxification [27,28]. Gut microbes also aid in resilience to pesticides by facilitating expression of cytochrome p450 enzymes involved in detoxification [29,30], so disruptions to the microbial community can have consequences for bee survival. However, evidence for the impact of pesticides on the gut microbiome is mixed. In some cases, chronic exposure to insecticides reduced the abundance of symbionts, perturbing the gut microbiome in commercial colonies of *Bombus terrestris* [31] but not *B. impatiens* [32]. Similarly, herbicides have been shown to disrupt the microbiome of managed bumble bees [33] but in other cases, impacts have not been demonstrated [34]. To our knowledge, no impact of fungicides has been demonstrated on the bumble bee bacterial microbiome [35,36], but impacts have been shown in other non-honey bee species [37].

How these disparate findings translate to wild bee populations remains uncertain, because much of our knowledge is derived from studies on commercial or managed bee colonies that have lower richness, diversity, and abundance of gut microbes than their wild counterparts [38]. Although there have been efforts to examine pesticide sensitivity in wild species [35], the degree to which population-level differences influence sensitivity to pesticides is not often considered. This is crucial because stressors that influence bee abundance vary across landscapes [39], and pesticide tolerance interacts with these stressors like parasite load [40,41], nutritional status [40,42] and even temperature [43,44]. In addition, wild bumble bees can exhibit variation in abundance of gut microbes across landscapes [45,46], likely due to variation in these stressors [47] and floral resources. If we want to identify generalizable patterns regarding which populations of wild bees (or their microbiomes) are most vulnerable to disruption by agricultural chemicals, a deeper understanding of factors driving sensitivity across landscapes is thus essential.

We aimed to understand spatial variation in sensitivity of a common, wild bumble bee species (*Bombus vosnesenskii*) to common pesticides including an insecticide, a fungicide, a herbicide and the combination of all three chemicals. We asked whether and how these chemicals affect survival and gut microbes, with a particular emphasis on the potential for site-specific effects.

## 2. Methods

### 2.1. Sites and field collection

**Figure 1.**
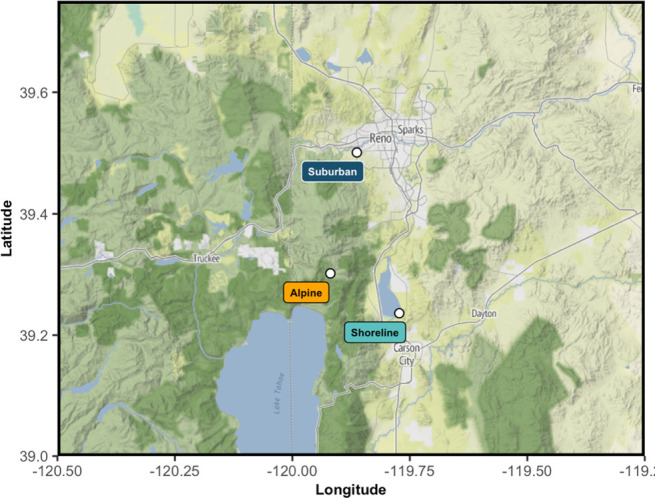
Bees were collected from an alpine meadow (gold), a scrubland shoreline (light blue) and a suburban park (dark blue) in northern Nevada.

Bees were collected from three sites in northern Nevada (figure 1) between 21 June 2022 and 5 July 2022 hereafter referred to as: an alpine meadow, a scrubland shoreline and a suburban park. Sites ranged from 14.58 to 30.46 km apart and differed in elevation and degree of urbanization (electronic supplementary material, table S1) [48]. To reduce bias in timing of collections, we rotated between collection sites over the 14-day collection period until we reached our target of 50 individuals per site. We collected *B. vosnesenskii* workers (*n* = 175); for consistency, we collected bees while foraging from either *Penstemon strictus*, *Penstemon heterodoxus*, or *Melilotus alba* and then transferred them to the labratory on ice.

### 2.2. Pesticide residue screening

We expected sites to vary in environmental pesticide exposure. To quantify site-level environmental pesticide exposure, we collected 10 of the last, fully expanded and undamaged leaves from five different individual *P. strictus*, *P. heterodoxus* and *M. alba* as available at each site (electronic supplementary material, table S2). Clippers were sterilized with bleach and ethanol between cuttings. Samples were stored in sealed plastic bags at −80°C until they were shipped to the Agriculture and Food Lab at the University of Guelph and screened for the presence of 510 agricultural chemicals (including those used in treatments described below: imidacloprid, tebuconazole and glyphosate).

All sample analyses were performed at the ISO/IEC 17025 accredited Laboratory Service Division, University of Guelph. The multi-residue pesticide determination was performed by liquid chromatography/electrospray ionization–tandem mass spectrometry (LC/ESI-MS/MS) and gas chromatography–tandem mass spectrometry (GC-MS/MS) on homogenized samples. Pesticides were extracted using the quick, easy, cheap, effective, rugged and safe (QuEChERS) method with dispersive solid phase extraction. A representative sample was extracted into acetic acid in acetonitrile in the presence of anhydrous sodium acetate and magnesium sulfate. The supernatant was split, evaporated and diluted with either methanol/ammonium acetate (for LC analysis) or acetonitrile (for GC analysis). Sample extracts were analysed in positive mode using a SCIEX 5550 ESI-MS/MS with Agilent 1260 HPLC and an Agilent GC Quadrupole 7890A GC-MS/MS. Additionally, samples underwent a modified version of the EU’s QuPPE method. Briefly, a representative subsample (with 5 ml of Nanopure water to rehydrate) was extracted using acidified (with formic acid) methanol and then placed into a freezer for 30 min. The supernatant was centrifuged, filtered and analysed in negative mode using a SCIEX 5500 LC-MS/MS with an Agilent HPLC. All methods used several quality control points that confirmed the presence of a compound in the sample: retention time (RT), M + 1 target mass, 2 fragment qualifier ions and the ratio of the two fragment ions. For the identification and quantification of the compounds, each method utilized deuterium-labelled internal standards, matrix-matched blanks, spikes and calibration curves.

### 2.3. Pesticide diets

In the laboratory, bees were transferred to individual experimental chambers (electronic supplementary material, figure S1): a sterile 50 ml Falcon tube with ventilation holes drilled along the sides. The cap of the tube was affixed with a 1.5 ml microcentrifuge tube feeder with a small hole at the tip fitted with a tapered cotton swab (Fran Wilson Nail Tees Cotton Tips) to prevent spillage and allow ad libitum access to the feeders.

We assigned bees to one of four experimental sucrose solution diets (herbicide, insecticide, fungicide, combination, or control). The herbicide treatment was 30 ppm of glyphosate (Sigma-Aldrich, USA), the fungicide treatment was 30 ppb of tebuconazole (Sigma-Aldrich, USA), and the insecticide treatment was 30 ppb of imidacloprid (Sigma-Aldrich, USA). These chemicals were selected because they had the highest estimated use for each chemical class (insecticide, herbicide, fungicide) in our region (Washoe county, Nevada) [49]. These concentrations of imidacloprid and tebuconazole approximate the median of those detected in nectar [11]. The concentration we chose for glyphosate is within the range of field-realistic exposure from treated plants [50–52] although is likely higher than bees would experience foraging from *non*-target plants [53]. We created a 1 : 1 stock solution for each focal chemical, which we then pipetted into working solutions of 30% (w/w) autoclave-sterilized sucrose (fresh working solutions were made weekly).

### 2.4. Survival assay and dissection

We monitored survival daily for 20 days. Every 3 days, we replaced feeders with a clean feeder with 1000 µl of solution. We continued the experiment until bees died or at day 20 (whichever came first) at which point we weighed the bees, measured their intertegular (IT) span to estimate body size [54] and dissected out the midgut and hindgut (hereafter ‘gut’) for DNA extractions and 16S sequencing (see below). We performed dissections under a fume hood and near an open flame, we also noted the presence of any parasitoid larvae. Bee guts and bodies were stored in separate microcentrifuge tubes at −80°C until DNA extraction.

### 2.5. DNA extractions, 16S sequencing, qPCR and bioinformatics

Microbial DNA was extracted from gut samples, using a ZymoBIOMICSTM DNA Miniprep Kit with Lysis Tubes following the manufacturer’s protocol. The resulting DNA was subjected to V4 16S rRNA sequencing using a previously described dual-indexed barcoding strategy [55] with recent modifications to the amplification sequences [56,57]. Amplicons were generated in a HEPA-filtered laminar flow cabinet dedicated to PCR preparation and decontaminated before and after use. Kit and reaction controls were also included in downstream sequencing. Reactions were carried out using 200 nM of each primer, 0.5 mM added MgCl_2_, and GoTaq Master Mix (Promega; Madison, WI, USA) in 25 μl volumes with the following programme in an MJ Research PTC-200 thermocycler: 94°C for 3 min, 25 cycles of 94°C for 45 s, 50°C for 60 s and 72°C for 90 s, followed by a final extension at 72°C for 10 min. PCR reactions were pooled and purified using a High Pure PCR purification kit (Roche) and paired-end sequencing (300 bp) was performed on an Illumina MiSeq at the Idaho State University Molecular Research Core Facility (RRID:SCR_012598).

From this extracted DNA, we evaluated total bacterial load (hereafter referred to as bacterial abundance) with SYBR-based qPCR to quantify bacterial copy number using the same primers as above. All samples were run in triplicate wells. Run conditions were 3 min at 95°C, followed by 40 cycles of 95°C for 15 s and 50°C for 60 s. Fluorescence at 520 nm was measured at the end of each cycle. The log copy number per ng DNA and relative abundances were calculated using standard curves of a plasmid (electronic supplementary material, figure S2). This plasmid standard was prepared from *Snodgrasella alvi* 16S gene, which has a complete reference genome and was detected across 43% of samples in our 16S sequencing.

We used QIIME2-2023.2 [58] to demultiplex FASTQ files and process the 16S rRNA gene sequence libraries. First, we trimmed primers and low-quality ends off reads with the DADA2 plug-in [59] and clustered sequences into amplicon sequence variants (ASVs). A phylogenetic tree was generated by aligning the sequences using the alignment MAFFT plug-in in QIIME2, masking highly variable positions and creating the tree via the FastTree plug-in [60]. The tree was rooted by implementing the phylogeny midpoint root plug-in and assigned ASV taxonomy using the SILVA database [61]. We filtered out ASVs from the resulting table that were assigned as chloroplast, eukaryotes, mitochondria, or that were not identified to at least phylum. After filtering, samples were rareified to 600 read pairs per sample to maximally balance sample inclusion and estimates of alpha diversity.

### 2.6. Statistical methods

All statistical analyses were carried out in R version 4.2.2 [62]. We modelled conopid presence (yes or no) as a factor of site, bee body size, microbiome community structure (PC1, see below) and microbial abundance using a generalized linear model with a binomial error structure. Because site and collection date covaried, collection date was modelled in a separate binomial model. We used the DHARMa 0.4.6 package [63] to test for uniformity and overdispersion of the residuals in all models, as well as outliers in the data. We found no evidence of these assumptions being broken in our models. To assess the impacts of treatment on survival, we created two separate linear models: a binomial regression to compare if a bee survived (yes or no), and a negative binomial generalized linear model using the glm.nb function from the MASS package [64] to compare the day a bee died (1–20) among the bees that died over the course of the experiment. These survival models included site, treatment, a site-by-treatment interaction, parasitoid presence (yes or no) and body size as predictor variables. We assessed model fit with type III ANOVA using the ANOVA function from the car package [65] and model assumptions with DHARMa as above.

To assess microbiome community structure, we generated a weighted UniFrac distance matrix in QIIME2 and visualized differences with principal coordinates analysis (PCoA). The PC1 axis from this was used as predictor variable in our survival model. We tested differences in community composition across sites, treatments, parasitoid presence (yes or no), body size, day a bee died and the site-by-treatment interaction using PERMANOVA from the vegan package [66]. We also used the package vegan [66] to estimate alpha diversity with inverse Simpson’s index (1/*D*). These metrics were used as response variables in separate generalized linear models with the same predictor variables as above. Finally, we modelled abundance using qPCR-corrected amplicon data of the top bacterial taxa using the same predictor variables as above. Again, we assessed model fit with the ANOVA function and verified model assumptions with the DHARMa package (see above).

## 3. Results

### 3.1. Site-level pesticide exposure

The chemical screening of focal plant vegetative tissue only detected three out of 510 chemical contaminants, two of which (chlorate and perchlorate) are naturally occurring and can be detected in high concentrations in arid environments [67]. The third chemical detected (*ortho-*phenylphenol or 2-phenylphenol) was only detected in trace amounts at the suburban park and alpine meadow (electronic supplementary material, table S2). Given this, we conclude that sites did not generally differ in their risk of agricultural chemical exposure via these specific plant species.

### 3.2. Conopid parasitoids and body size

In total, 14.9% of bees were found to have conopid parasitoid larvae. The shoreline site had the highest incidence of parasitoids (21 of 73 bees, 29%), with fewer at the alpine site (8 of 60 bees, 13%) and none at the suburban site (0 of 65 bees, 0%). The presence of conopid parasitoids was best predicted by site and collection date (*X*^2^ = 14.06, *p* < 0.001 and *X*^2^ = 6.22, *p* = 0.01, respectively) with the majority of conopid parasitized bees being collected on later dates. Other factors (body size and microbiome structure and abundance; electronic supplementary material, table S4) were not important predictors of conopid presence. However, our bacterial abundance model suggested a reduction in gut bacterial abundance with the presence of conopids (see below).

Bees from different sites varied in body size (*X*^2^ = 46.33, *p* < 0.001). Bees from the alpine site were an average of 8.2% larger than bees from the shoreline site, and an average of 11.2% larger than bees from the suburban site. Shoreline bees were only an average of 3.0% larger than our suburban bees.

### 3.3. Survival

**Figure 2.**
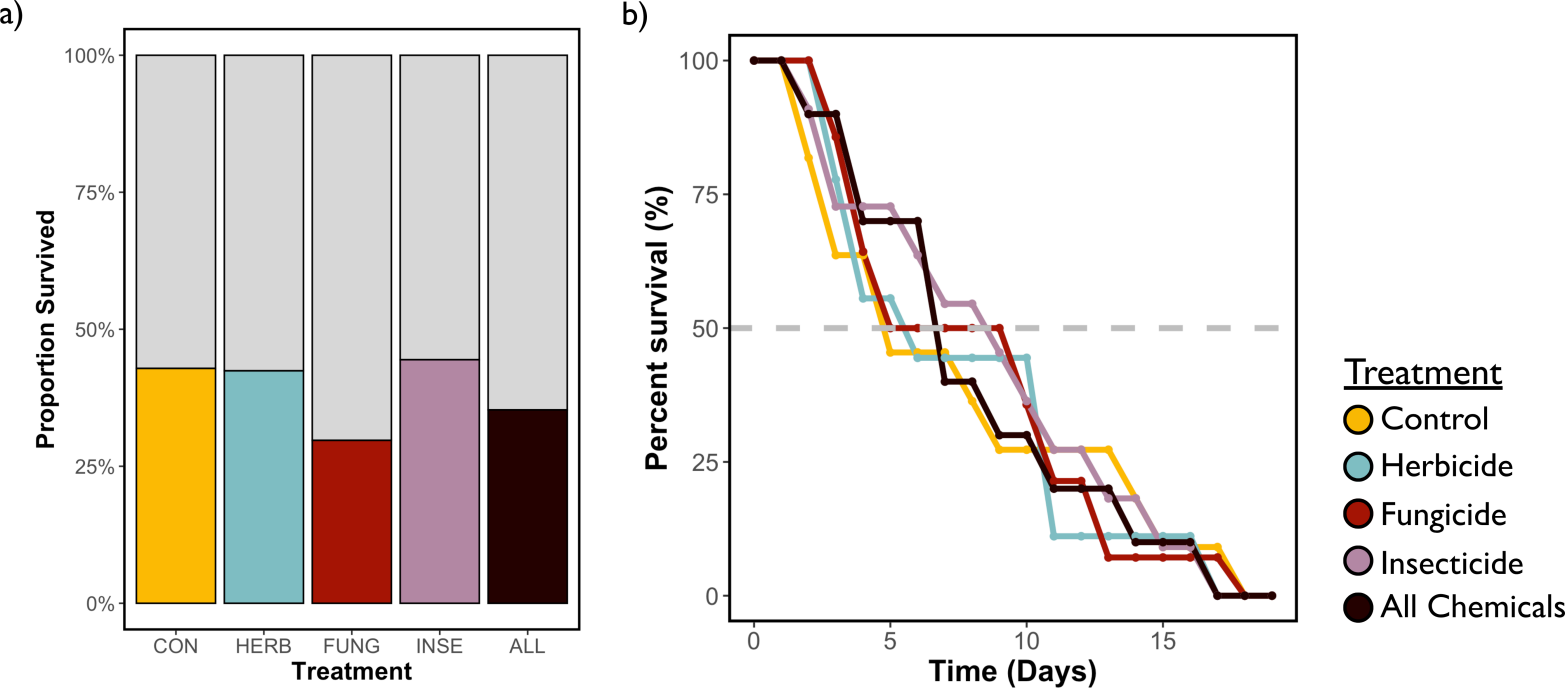
Proportion of bees that survived the experiment by pesticide treatment (CON = control, HERB = herbicide, FUNG = fungicide, INSE = insecticide, and ALL = combination of all chemicals) (a) and percent survival of bees that died over the course of the experiment by pesticide treatment (b) over the course of the 20-day experiment. A dashed grey line is included at 50% survival.

Conopid presence and body size were the best predictors of whether a bee survived the course of the experiment (table 1; conopid: *X*^2^ = 26.73, *p* < 0.001, body size: *X*^2^ = 11.29, *p* < 0.001). Of the bees without parasitoids, 47.5% survived compared to 2.9% of parasitized bees. Interestingly, smaller bees were more likely to survive the experiment, with those that survived having an average IT span that was 2.0% shorter than those that did not. Whether a bee survived did not vary by treatment nor was there a site-by-treatment interaction (figure 2).

**Table 1.** Summary table of model outputs of the binomial response variable survived (y/n) or the day a bee died by site, conopid presence (y/n) body size (IT space), treatment and the treatment-by-site interaction. *p < 0.05; **p < 0 .01; ***p < 0.001.

survived (y/n)	*X* ^2^	d.f.	*p*‐value	
conopid (y/n)	26.73	1	<0.001	***
body size (IT)	11.29	1	<0.001	***
site	2.34	2	0.31	
fungicide	0.14	1	0.71	
insecticide	0.93	1	0.34	
herbicide	0.04	1	0.85	
all chems	0.00	1	0.97	
site × fungicide	0.37	2	0.83	
site × insecticide	2.36	2	0.31	
site × herbicide	0.54	2	0.76	
site × all chems	0.23	2	0.89	

day died				
conopid (y/n)	7.14	1	<0.001	**
body size (IT)	0.72	1	0.40	
site	15.00	2	<0.001	***
fungicide	2.21	1	0.14	
insecticide	0.99	1	0.32	
herbicide	0.55	1	0.46	
all chems	2.89	1	0.09	
site × fungicide	9.63	2	<0.01	**
site × insecticide	2.63	2	0.27	
site × herbicide	11.99	2	<0.01	**
site × all chems	12.33	2	<0.01	**

**Figure 3.**
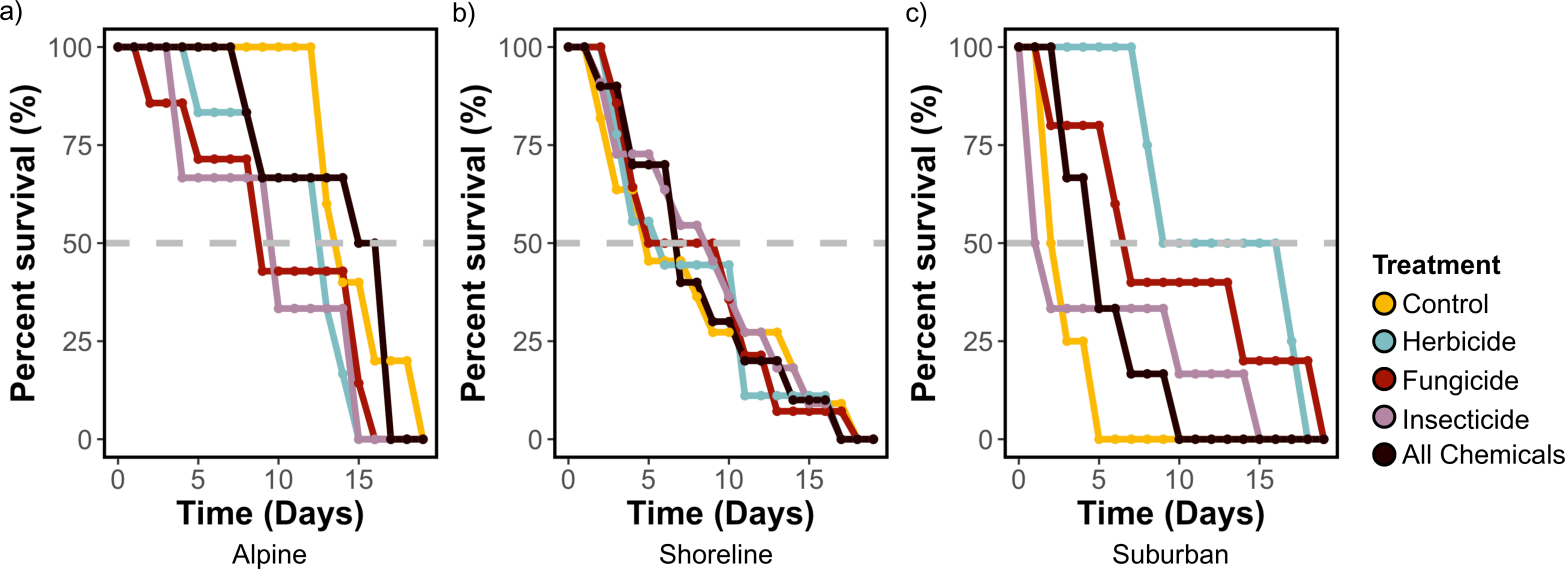
Percent survival of bees that died over the course of the experiment by pesticide treatment at the alpine site (a), shoreline site (b) and suburban site (c) across the 20-day experiment. A dashed grey line is included at 50% survival.

Among bees that died over the course of the experiment (*n* = 107), conopid presence was again an important predictor of the day a bee died (*X*^2^ = 7.14, *p* < 0.01) and bees without a conopid survived an average of 1.9 days longer than those with a conopid. Site was again an important predictor of the day a bee died (*X*^2^ = 15.00, *p* < 0.001), with the average alpine bee surviving to day 12.1, the average shoreline bee surviving to day 8.1 and the average suburban surviving to day 7.0. Here, we also saw evidence of treatment effects. Bees assigned a diet containing the combination of all three chemicals showed marginally shorter survival time than control bees (figure 2b; *X*^2^ = 2.89, *p* = 0.09), but this was the only treatment that had an overall impact on survival. However, we did observe a site-by-treatment interaction with the fungicide, herbicide and all chemical treatments (figure 3a–c). Briefly, alpine bees survived longer than bees from the other two sites in every treatment (electronic supplementary material, tables S5; 2–12 days longer on average, varying with treatment) except the herbicide treatment, where suburban bees survived 2 days longer than alpine bees.

### 3.4. Bacterial abundance and microbiome structure

**Figure 4.**
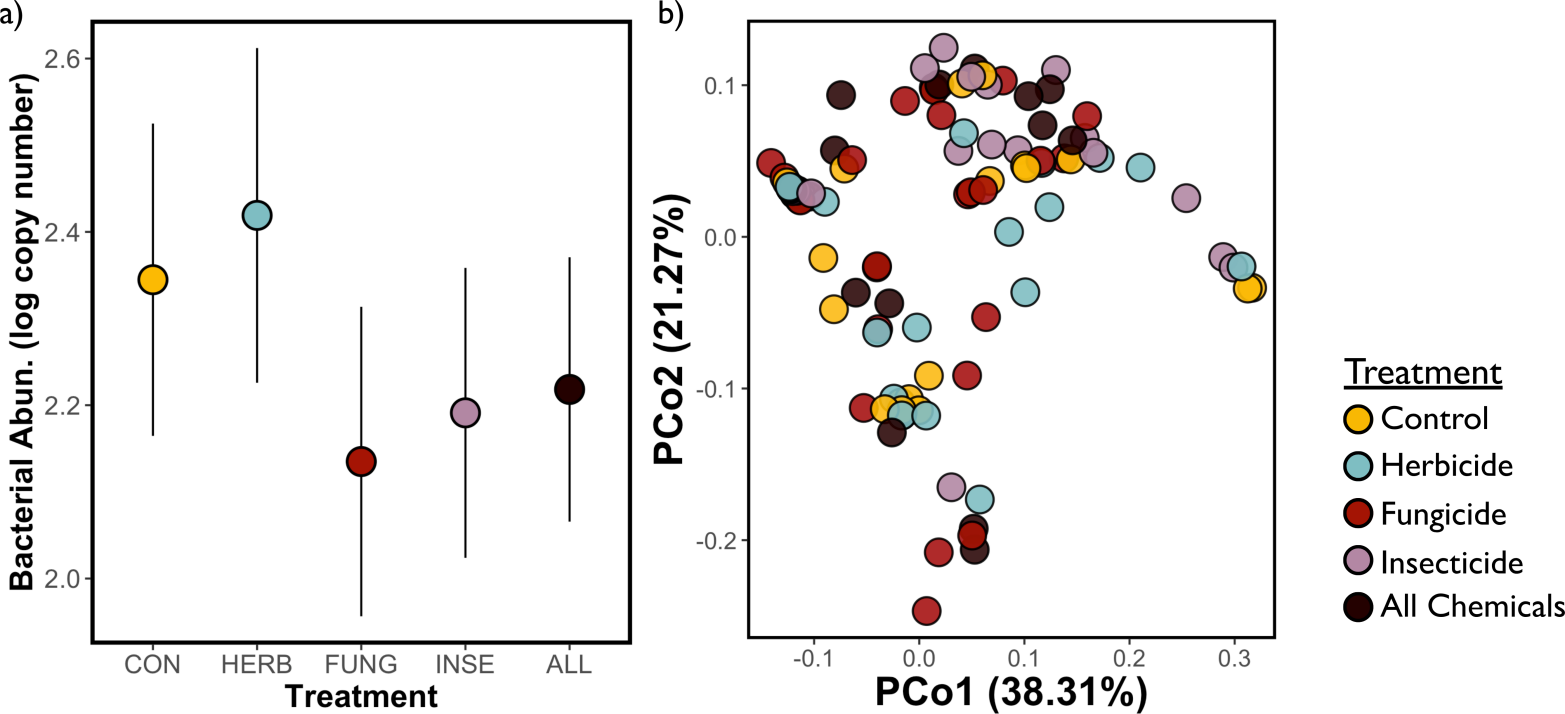
(a) Mean bacterial abundance (log copy number) denoted with circles, ± s.e. in bees that received control diet (CON), herbicide (HERB), fungicide (FUNG), insecticide (INSE) and the combination of all chemicals (ALL). (b) Microbiome composition across treatment with loadings from principal coordinates analysis (PCoA).

We were able to successfully estimate bacterial abundance for all 175 individuals. Bacterial abundance differed by conopid presence and body size (electronic supplementary material, table S6; *X*^2^ = 3.85, *p* = 0.05 and *X^2^* = 4.72, *p* = 0.03, respectively). Bees that were parasitized by a conopid had an average of 13.8% fewer bacteria copies compared to those that were unparasitized and larger bees had more gut bacteria. We did not find strong differences by treatment (figure 4a) or any of our other variables of interest in predicting the abundance of gut bacteria in these bees.

After quality filtering and rarefaction, 114 samples remained for microbiome structure analysis (43 alpine bees, 54 shoreline bees and 17 from the suburban site). Of these samples, we also did not find evidence that gut microbiome community composition differed by treatment (figure 4b). Although, insecticide-treated bees had marginally higher richness than control bees (*X*^2^ = 4.99, *p* = 0.03). Insecticide-treated bees did not differ in diversity metrics by site (electronic supplementary material, table S7), but we did see taxon-specific responses to insecticide (see below). We observed site-level differences in community composition (electronic supplementary material, figure S3; *F* = 2.36, *p* = 0.02) and we also observed difference in gut community composition depending on if bees survived longer over the course of the experiment (*F* = 3.29, *p* = 0.01). Again, we did not observe differences in diversity metrics in bees that survived longer in the experiment (electronic supplementary material, table S7), so this difference is likely driven by taxa-specific differences in abundance (see below).

We also found inverse Simpson’s diversity and richness were higher in larger-bodied bees (*X*^2^ = 6.25, *p* = 0.01 and *X*^2^ = 3.69, *p* = 0.05, respectively) and bees with a conopid had marginally lower evenness (*X*^2^ = 3.69, *p* = 0.05). The top five most abundant bacterial taxa in the bee gut were *Schmidhempelia*, followed by *Bombiscardovia*, *Snodgrassella*, *Enterobacter* and *Gilliamella*, but only *Schmidhempelia* had enough representation across samples to be included in further analysis and, even then, we had the most confidence in our binomial model. *Schmidhempelia* was more likely to be present in larger-bodied bees (*X*^2^ = 7.21, *p* < 0.01), and we found marginal evidence for the presence of *Schmidhempelia* with the site-by-insecticide interaction (*X*^2^ = 5.21, *p* = 0.07).

## 4. Discussion

In this study, we aimed to understand intraspecific variation in a wild bumble bee’s sensitivity to multiple pesticides. We found that, regardless of the site, bees showed a marginal reduction in survival when given our chemical combination treatment. Yet, site of origin played a strong role in determining a bee’s survival. We found evidence for a site-by-treatment interaction influencing the day a bee died across all pesticide treatments, except the insecticide group. The microbiome did not differ when bees were exposed to the pesticide treatment, within or across sites. Instead, gut microbiome differences were associated with the presence of conopid parasitoids and body size, both of which varied by site. These findings suggest that site-specific factors influence pesticide sensitivity and should be considered in ecotoxicological studies of wild bees.

### 4.1. Survival and parasitoids

The presence of conopid parasitoids significantly reduced bee survival regardless of treatment. Conopid flies (*Physocephala* sp.) are obligate endoparasitoids of bumble bees whose larvae feed on bee haemolymph and kill the host just before pupation, 10−12 days after infection [68–70]. Pupae are morphologically distinct from larvae and develop within 24 h of host mortality [69,70], and although we did not find pupae in our bees, it is possible the mortality we observed in our bees was the direct result of conopid larvae. Conopids were not well represented across treatments, so we were unable to ask questions about the impacts of pesticides on conopid–bee interactions. Yet a handful of studies do demonstrate interactive effects of pesticides and parasites (namely, *Crithidia bombi*) in bumble bees [40,71]. We hope future studies will explore the potential of agricultural chemicals, and insecticides specifically, to either disrupt or enhance bee–parasitoid dynamics.

Body size also played a significant role in survival, regardless of treatment. Smaller bees were more likely to survive than larger individuals, possible due to lower energy requirements and reduced food intake [72], which might limit pesticide exposure. Yet the largest bees survived the longest, perhaps due to their greater ability to handle stressors like pesticide exposure [73,74] or parasitoid presence, or other laboratory-based stressors like isolation from a colony or our artificial nectar solution.

Site also predicted timing of mortality over the course of the experiment, and we found evidence for treatment effects on survival that varied by site. Suburban bees had the poorest survival overall, possibly due to exposure to uniquely urban factors including light pollution, competition with non-native honey bees (which were only observed at our suburban site), or heavy metals that have been shown to impact bee physiology in urban environments [75]. Curiously, suburban bees had the lowest survival on the control solution and the highest survival on the herbicide treatment. One possible explanation for this is a priming effect of past exposure from adjacent neighbourhoods. Although we did not detect any glyphosate in our pesticide residue screening of leaves, *B. vosnesenskii* can forage up to 1867 m [76] and glyphosate can linger in plant tissues for years [11,77]. Given the different degrees of urbanization across sites, it is somewhat surprising that pesticide residue analysis showed no differences in the focal plant tissues we compared. Past studies have shown that leaves from suburban areas can have the same diversity of pesticide contamination as other landscapes, and certain compounds may be more highly concentrated [10]. However, we note that it is possible that certain fat-soluble pesticides may have been detected in the pollen load of bees, and there are other possible routes of exposure for ground-nesting bees such as *Bombus*, including the soil [78,79], that may not have been represented in our screening. In contrast, shoreline bees had intermediate survival and exhibited consistent survival rates regardless of treatment, likely due to added stressors like conopid parasitism. Alpine bees exhibited the highest overall survival, but their reduced survival when exposed to insecticides aligns with previous research suggesting that insecticides pose the greatest risk to bees [22,80]. Here, we demonstrate that bee response to pesticides can be context-dependent with landscape-level factors like parasitoid presence and nutritional status likely playing a strong role.

### 4.2. Gut microbes

We did not find strong evidence that exposure to these chemicals changes the total abundance or composition of the gut bacterial community in these bees, consistent with select previous studies of bumble bees [32,34,35]. To our knowledge, the only other study examining the impacts of pesticides on wild bumble bee microbiomes comes from Rutkowski *et al*. [35], who also found no impact of a fungicide treatment on gut bacterial composition. Together, these results might imply a certain robustness in the gut microbiome of wild bumble bees, but these results should be interpreted with caution. Because bees can be exposed to a wide range of pesticide concentrations in the field [11,53], future studies should explore possible impacts of higher concentrations on the microbiome of wild bees. In our study, insecticide-, fungicide-, and combination-treated bees had lower bacterial abundance than control bees (although this difference was not significant). Previous work in honey bees has shown microbe response to pesticides can be more severe at higher concentrations, but this is not true for all pesticides [81], so we caution that further study may be required to draw conclusions about the impacts of pesticides on the wild bee gut microbiome.

We found a change in the gut community in bees that survived longer, which is consistent with other studies demonstrating a change in gut bacterial communities over time [82]. We also observed site-level differences in gut community structure wherein our suburban site differed from the other two sites, and *post hoc* analysis showed this site had fewer gut bacteria (electronic supplementary material, table S3). Bees from this site also had the poorest overall survival. Past laboratory-based experiments of microbe-depleted commercial bumble bees have not shown similar reductions in survival [83]. Either the microbiome of wild bees is more tightly linked to survival, or other site-level factors like poorer nutritional status contributed to both the gut health and survival of these bees. We hope future studies will examine this relationship more closely, perhaps using shotgun sequencing or similar approaches.

Finally, we found that conopid presence reduced the total abundance of gut bacteria. This is likely simply because conopids occupy the abdominal cavity and reduce the gut volume [84]. So, in addition to the direct, negative health consequences of conopid parasitism, conopid presence may have indirectly affected bee health by reducing the abundance of gut bacteria. However, the gut microbiome did not influence the probability of conopid infection. Past work has shown the gut microbiome plays a role in protecting against microbial parasites and pathogens through the production of anti-microbial peptides (i.e. *Crithidia* sp., *Nosema* sp. and *Escherichia coli* [45,85,86]). Together this suggests gut microbes may not protect against insect parasitoids, but conopids or other parasitoids may still indirectly shift gut microbial function by reducing microbial abundance.

## 5. Conclusions

We tested field-realistic concentrations of common pesticides [11] that did cause site-specific mortality, yet it is essential to note that these chemicals can have serious sub-lethal effects such as impacts to immune response, metabolism, reproduction, cognition, and more [87,88]. Again, although the concentrations we used in this study did not cause changes in gut bacterial abundance or composition, we urge caution applying these findings broadly to other insect taxa. The species used in this study is not currently thought to be threatened and in fact is increasing its range and abundance [89]. Other species such as solitary bees—whose gut microbes are environmentally acquired and vary greatly across landscapes—may respond differently [90–92]. Studying solitary species could provide valuable insight into how landscape-level factors shape microbiome composition [93] and pollinator health.

Finally, our findings highlight the critical need to focus on wild bee populations. With growing concern swarming around bee declines, it is essential to understand the factors driving these declines in real-world contexts. Site-specific factors, such as conopid prevalence and body size, had a clear influence on survival, emphasizing that pesticide sensitivity assessments must account for these variables to fully capture population-level patterns. Expanding research to include wild bees will allow for a deeper understanding of ecological complexities and help inform more effective conservation strategies.

## Data Availability

Sequencing data are available on the NCBI Sequence Read Archive (SRA) BioProject ID number PRJNA1221307. Additional datasets generated by this study have been deposited in the USDA Ag Data Commons [94]. The R script and files used for statistical analysis generated by this study are available at Zenodo [95]. Supplementary material is available online [96].
